# Supplementary material for: Membrane Interaction of Ibuprofen with Cholesterol-Containing Lipid Membranes
Source: Biomolecules. 2020 Sep 28;10(10):1384. doi: 10.3390/biom10101384 (PMC7650631; doi:10.3390/biom10101384)
Supplement: Supplementary file 1 [file biomolecules-10-01384-s001.zip › IBu Supporting information_final.pdf]

Supporting information

Membrane interaction of ibuprofen with cholesterol-containing lipid membranes

Jan Kremkow, Meike Luck, Daniel Huster, Peter Müller, Holger A. Scheidt

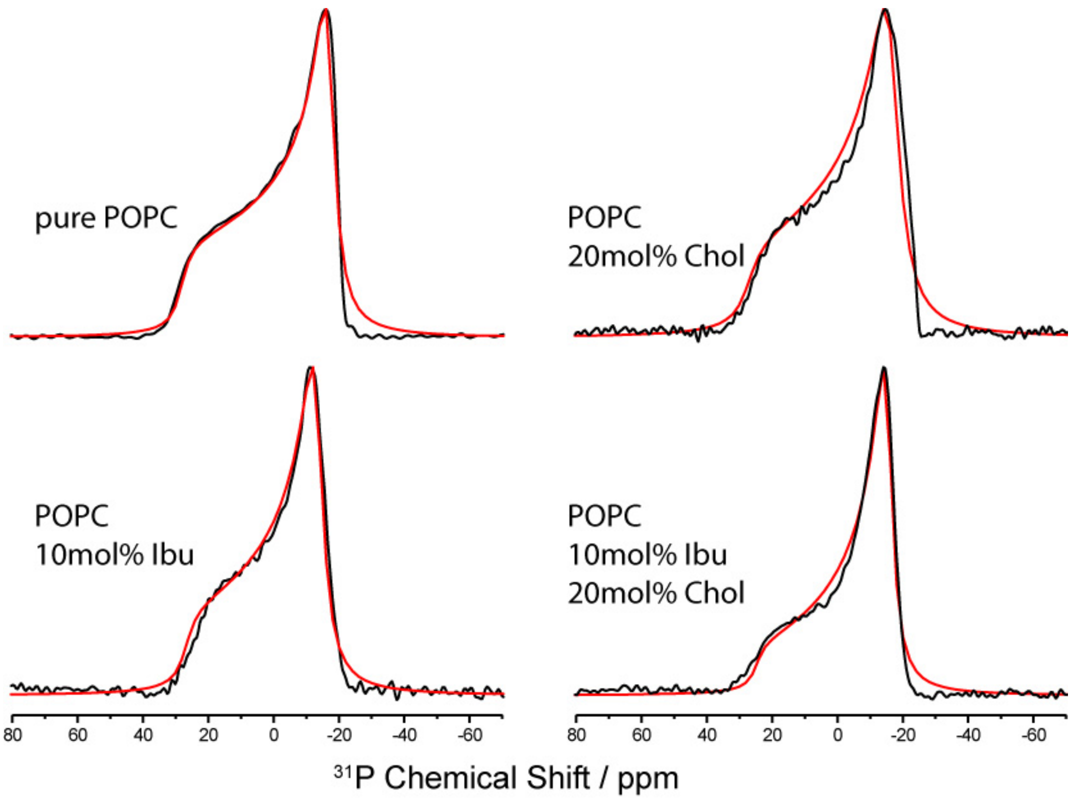

Supplementary Figure S1: ^{31}P NMR spectra (black) of POPC-d_{31} membranes in the presence of Ibuprofen and/or cholesterol and the respective numerical simulation of the experimental spectra (red).

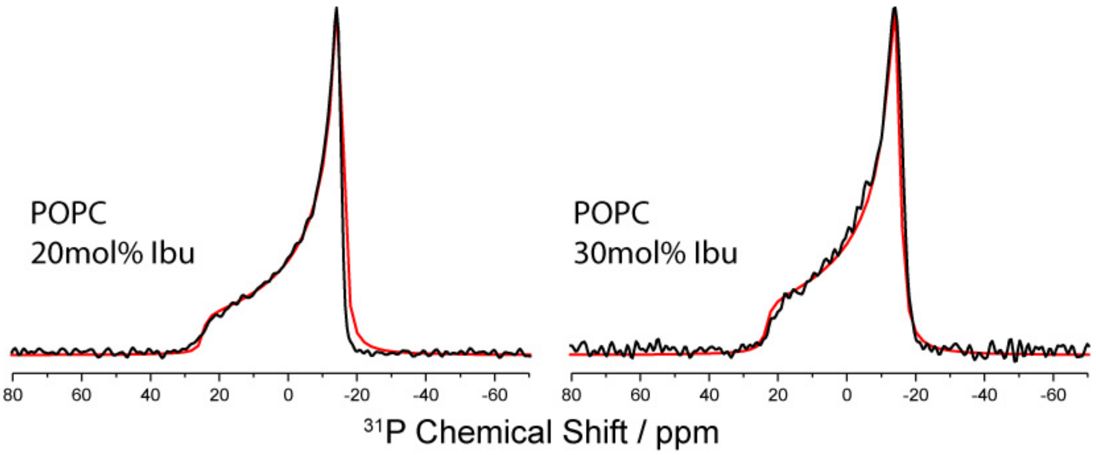

Supplementary Figure S2: ^{31}P NMR spectra (black) of POPC-d_{31} membranes in the presence of 20 mol% (left) and 30 mol% (right) ibuprofen and the respective numerical simulation of the experimental spectra (red).

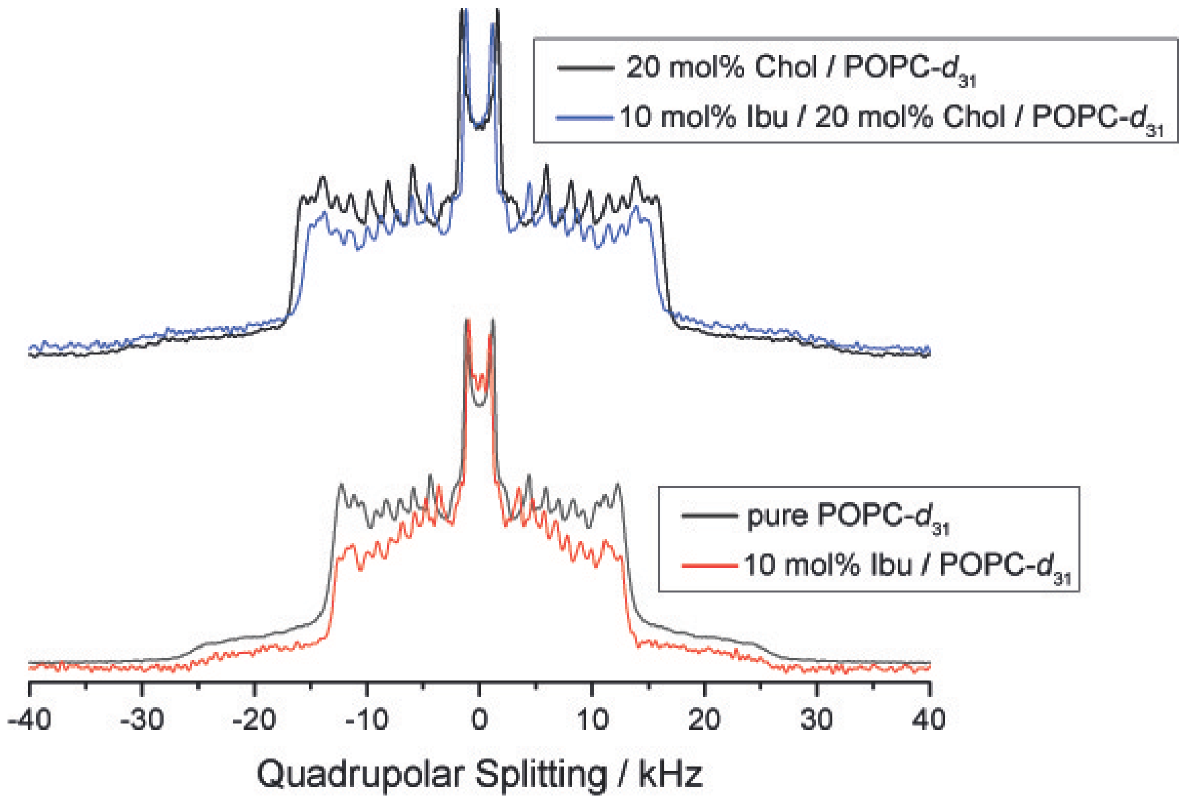

Supplementary Figure S3: Influence of 10 mol% Ibuprofen on the ^{2}H NMR spectra of POPC-d_{31} membranes (bottom) and POPC-d_{31}/cholesterol (80/20, mol/mol) membranes (top).

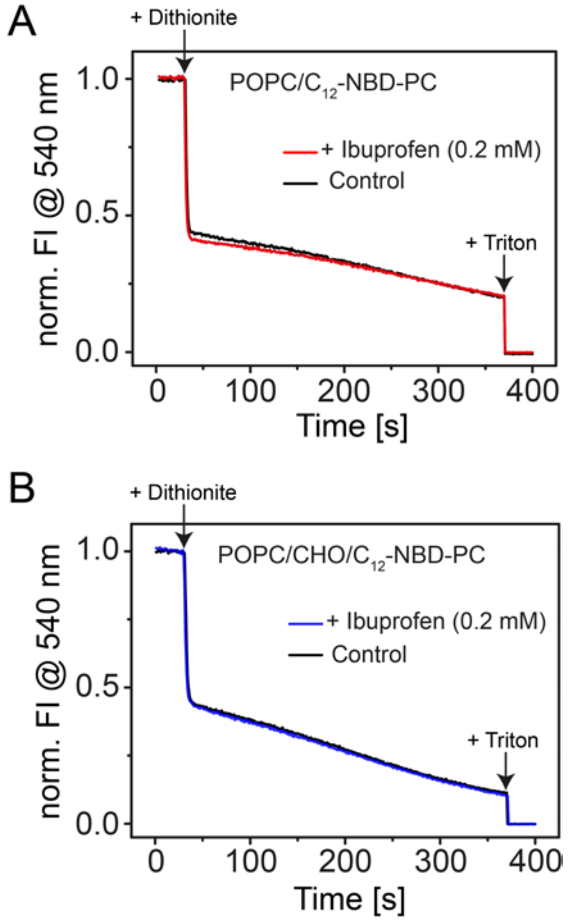

Supplementary Figure S4: Influence of ibuprofen on the dithionite-mediated reduction of NBD-PC in POPC LUVs (A) and POPC/Chol (80/20, mol/mol) LUVs (B). Vesicles (lipid concentration 1 mM, 0.5 mol% NBD-PC) were mixed in a cuvette with 0.2 mM ibuprofen or ethanol vehicle (at the same volume as added with the drug). Subsequently, 1.45 ml HBS50 (Hepes buffered saline, 150 mM NaCl and 50 mM Hepes, pH 7.4) was added and fluorescence was monitored with time at 540 nm (λ_{ex} = 470 nm) at 37°C. At t = 30 s, sodium dithionite was added (final concentration 50 mM) and at t = 360 s Triton X-100 (0.5 w/v%) to solubilize the vesicles resulting in complete reduction of fluorescence. The curves were normalized to the fluorescence intensities before addition of dithionite.

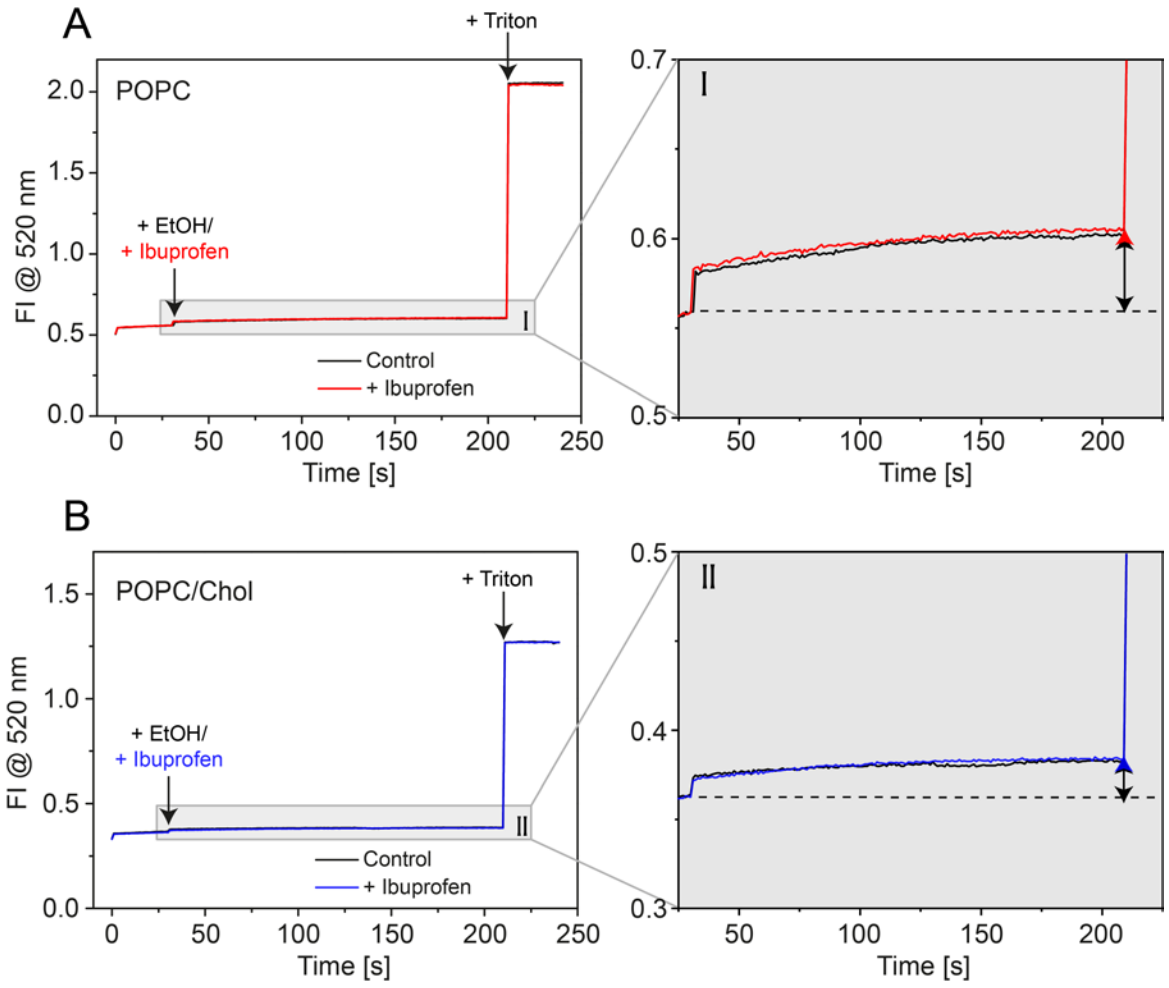

Supplementary Figure S5: Ibuprofen-induced leakage of carboxyfluorescein (CF) from POPC LUVs (A) and POPC/Chol (80/20, mol/mol) LUVs (B) and enlarged views of the slow rise of CF fluorescence intensity in POPC LUVs (I) and POPC/Chol LUVs (II). Vesicles entrapping CF were prepared. 130 µl CF-containing LUVs were mixed with 1.43 ml HBS and given to a fluorescence cuvette. The time-dependent fluorescence was recorded at 520 nm (λ_{ex} = 490 nm; slit width for excitation and emission, each 4 nm) at 37°C. At t = 60 s, ibuprofen (molar ratio lipid/drug = 5) or ethanol vehicle (same volume as added with ibuprofen) was added, and at t = 210 s Triton X-100 (0.5 w/v%) to solubilize all vesicles. Two independent experiments were performed giving similar curves. The small increase of fluorescence intensity observed upon sample addition is probably caused by an impact of ethanol on (part of) the vesicles.

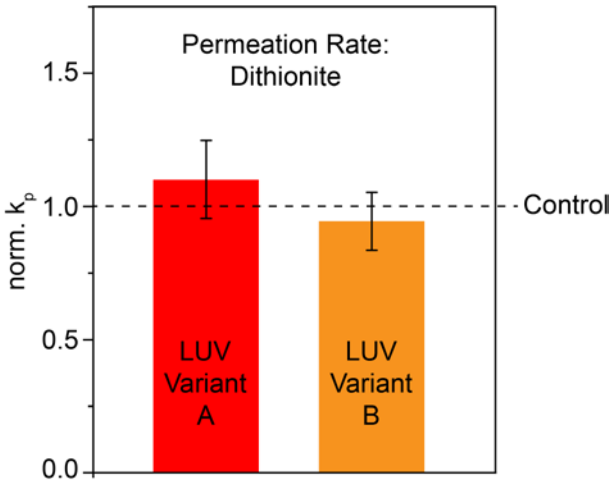

Supplementary Figure S6: Normalized rate constants (k_P) reflecting the permeation of dithionite across the membrane in POPC LUVs. Ibuprofen (molar ratio lipid/drug = 5:1) was added after the LUV preparation to the preformed membranes (LUV variant A; left) or was incorporated into the lipid membrane through the organic phase before bilayer formation like done for the NMR experiments (LUV variant B; right). k_P values determined in the presence of ibuprofen were normalized to those determined of respective membranes in the absence of the drugs. The vesicles contained 1 mM lipid and 0.5 mol% NBD-PC. The data represent the mean +/- SE of ≥ 6 independent experiments.

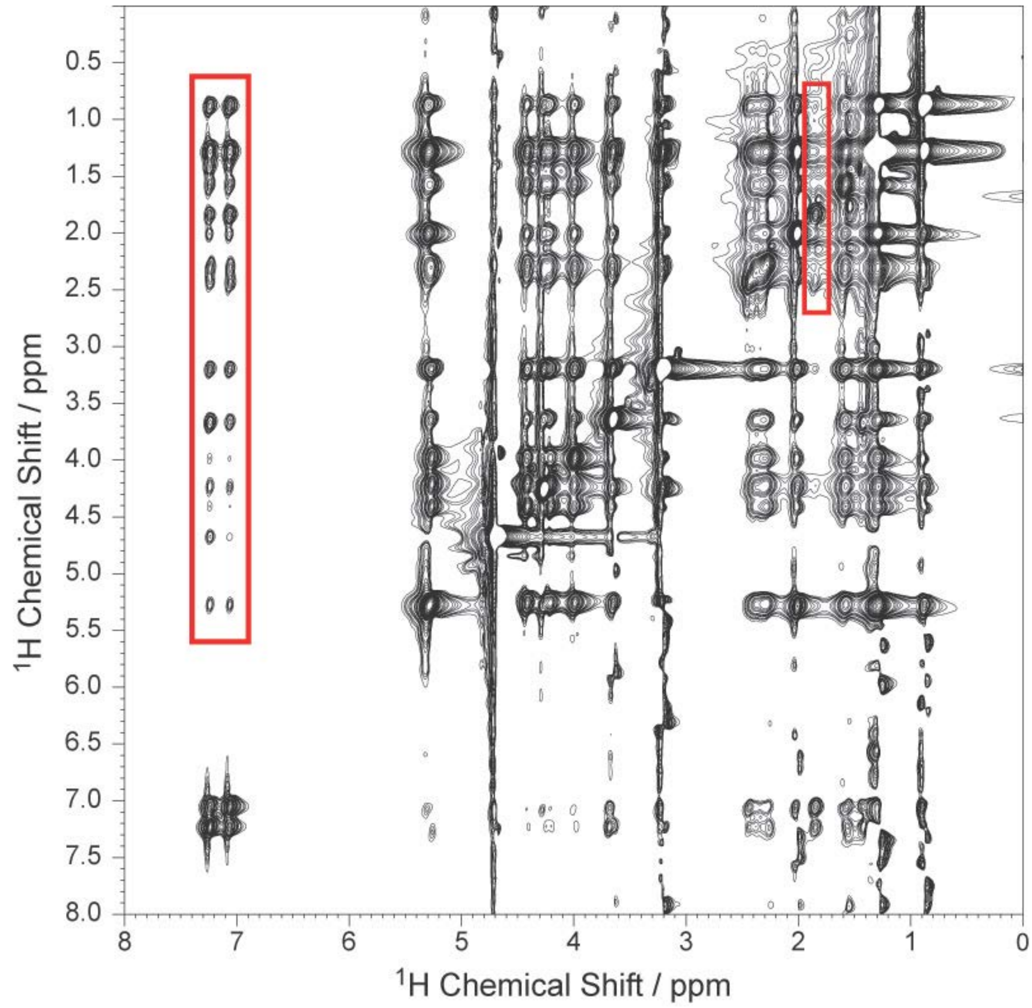

Supplementary Figure S7: ^{1}H NOESY MAS NMR spectrum of POPC membranes in the presence of 10 mol% ibuprofen. The regions with the cross peaks used for the quantitative analysis are highlighted. The spectrum was measured at a mixing time of 300 ms, a MAS frequency of 6,000 Hz and a temperature of 30°C.
